# Topological signatures of brain dynamics: persistent homology reveals individuality and brain–behavior links

**DOI:** 10.3389/fnhum.2025.1607941

**Published:** 2025-05-30

**Authors:** Yue Wang, Junxing Xian, Yuanyuan Chen, Yan Yan

**Affiliations:** ^1^Academy of Medical Engineering and Translational Medicine, Medical School of Tianjin University, Tianjin University, Tianjin, China; ^2^College of Precision Instruments and Optoelectronics Engineering, Tianjin University, Tianjin, China; ^3^State Key Laboratory of Cognitive Neuroscience and Learning, Beijing Normal University, Beijing, China; ^4^State Key Laboratory of Biomedical Imaging Science and System, Shenzhen Institutes of Advanced Technology, Chinese Academy of Sciences, Shenzhen, Guangdong, China; ^5^Center of Medical Information, Wenzhou Institute of Technology, Wenzhou, China

**Keywords:** functional magnetic resonance imaging, topological data analysis, persistent homology, individual differences, brain-behavior relationships

## Abstract

**Introduction:**

Understanding individual differences in brain dynamics is a central goal in neuroscience. While conventional time series features capture signal properties of local brain regions, they often fail to reveal the deeper structure embedded in the brain's complex activity patterns.

**Methods:**

Resting-state fMRI data from approximately 1,000 subjects in the Human Connectome Project were analyzed. A TDA-based framework integrating time-delay embeddings and persistent homology was employed to extract global dynamic features from resting-state fMRI data. Classification models and canonical correlation analysis (CCA) were employed to examine the associations between brain topological features and individual characteristics, including gender and behavioral traits.

**Results:**

Topological features exhibited high test-retest reliability and enabled accurate individual identification across sessions. In classification tasks, these features outperformed commonly used temporal features in predicting gender. Canonical correlation analysis identified a significant brain-behavior mode that links topological brain patterns to cognitive measures and psychopathological risks. Regression analyses across behavioral domains showed that persistent homology features matched or exceeded the predictive performance of traditional features in higher-order domains such as cognition, emotion, and personality, while traditional features performed slightly better in sensory-related domains.

**Discussion:**

These findings highlight persistent homology as a robust and informative framework for modeling individual differences in brain function, offering promising avenues for personalized neuroimaging analysis.

## 1 Introduction

Understanding individual differences in brain function is key to exploring healthy variations related to personality, gender, age, as well as advancing personalized treatments for neuropsychiatric disorders. Recent technological advancements have enhanced the sensitivity of fMRI, enabling the capture of individualized, refined brain states while minimizing noise interference (Vidaurre et al., 2017; Bitan, 2024). As a result, utilizing neuroimaging data to investigate these individual differences has become increasingly important and popular (Dubois and Adolphs, 2016; Huskey et al., 2020).

Previous studies have demonstrated that spontaneous brain activity during resting-state conditions can be used to predict a range of individual behavioral traits, including age (Gonneaud et al., 2021), gender (Ryali et al., 2024), cognition (Smith et al., 2015; Cabral et al., 2017; Liu et al., 2023), personality dimensions (He et al., 2024), and susceptibility to mental disorders (Li et al., 2020; Zhang et al., 2024; Aborode et al., 2025). Currently, the functional connectome-based (FC-based) approach has been widely adopted to characterize individual differences in brain function (Miranda-Dominguez et al., 2014; Finn et al., 2015; Finn and Todd Constable, 2016; Shen et al., 2017). In this framework, the rich temporal dynamics of fMRI signals are compressed into a static network representation, enabling a compact and interpretable summary of brain-wide functional organization. Studies have shown that these methods are robust and effective in capturing inter-individual variability, and they have been widely used to predict behavioral and clinical outcomes (Shen et al., 2017; Sui et al., 2020). However, FC-based methods have certain limitations. Specifically, FC relies on the assumption of linear, symmetric, and stationary interactions between brain regions, which may not fully reflect the non-linear and time-varying nature of neural processes (Hutchison et al., 2013; Lindquist et al., 2014). Moreover, by summarizing the entire time series into a single correlation value, FC discards potentially informative temporal features, such as transient dynamics, non-linearity, and phase relationships, which may carry unique individual-specific signatures (Lurie et al., 2020; Shafiei et al., 2020; Liu et al., 2023).

These limitations motivate the exploration of alternative representations that retain more of the intrinsic structure of brain dynamics. Therefore, temporal features extracted directly from the ROI time series have become another focus of attention (Zou et al., 2008; Garrett et al., 2010). These features aim to characterize the intrinsic statistical and dynamical properties of brain activity within each region, rather than inter-regional relationships. Commonly used metrics include variance, autocorrelation, self-predictability and various indicators of non-linearity and complexity, such as entropy or fractal dimension (Sokunbi et al., 2013; Fulcher and Jones, 2017; Lubba et al., 2019). Nevertheless, studies directly extracting features from time series also have inherent limitations. Many of the temporal features are manually crafted and depend on specific statistical assumptions, potentially limiting their generalizability across subjects or cognitive states. Additionally, while they retain more temporal information than FC, they still often overlook the underlying geometric or structural organization of the time series in its full high-dimensional space.

The human brain displays intricate and often chaotic temporal patterns, which present challenges for traditional analytical methods. To better characterize the non-linear, high-dimensional structure of brain dynamics, recent research has turned to Topological Data Analysis (TDA)—a mathematical framework designed to capture the intrinsic shape of data (Chazal and Michel, 2021). TDA enables the identification of topological features such as loops and voids, which describe how data points are organized in space and evolve over time. Unlike traditional statistics, these topological descriptors are invariant under continuous transformations and robust to noise, making them particularly well-suited for neural data (Caputi et al., 2021; Skaf and Laubenbacher, 2022).

Recent studies applying TDA to individual difference identification in small sample sizes have revealed that higher-order topological features may serve as stable individual-specific signatures (Santoro et al., 2024). Building upon this idea, we propose to apply persistent homology (PH)—a core method within TDA—to fMRI time-series data. We use over 1,000 subjects from the Human Connectome Project (HCP) dataset to extract temporal topological signatures from cortical ROI time series. These signatures exhibit clear individual specificity, suggesting their potential as functional fingerprints. To further explore the behavioral relevance of these topological representations, we employ canonical correlation analysis (CCA) to examine associations between individual topological signatures and multiple behavioral traits. Our results reveal strong and significant relationships between brain topology and behavior. Moreover, when compared to conventional temporal feature metrics, persistent homology demonstrates superior performance in both gender classification and behavioral prediction tasks, highlighting the advantage of topological approaches in capturing individualized brain dynamics.

## 2 Materials and methods

### 2.1 Dataset

This study uses resting-state fMRI data from 1,200 healthy adults (aged 22–36) provided by the Human Connectome Project (HCP) (Van Essen et al., 2012, 2013). The MRI data were acquired using a 3T Siemens Prisma scanner, with acquisition parameters detailed in the literature (Ugurbil et al., 2013; Van Essen et al., 2013). During the resting-state fMRI data collection, participants remained awake and rested quietly for 15 minutes. Each participant underwent two sessions (day 1 and day 2), each containing two scans with opposite phase encoding directions. The behavioral measurements used in this study were provided by the HCP dataset.

This study uses the minimally preprocessed fMRI data provided by the HCP (Glasser et al., 2013). The minimal preprocessing pipeline includes steps such as gradient distortion correction, motion correction, and concludes with non-linear registration to the MNI152 standard space. Then, the effects of head motion, temporal trends, cerebrospinal fluid signals, white matter signals, and global signals are regressed out. This study includes 1,013 individuals whose data were complete and successfully processed through the pipeline. A bandpass filter was then applied with a frequency range of 0.01–0.08 Hz. This study uses the Schaefer 200 atlas in the MNI152 standard space, which includes 200 regions of interest (ROI) divided into 7 brain networks (Schaefer et al., 2018).

### 2.2 TDA framework

**Figure 1:**
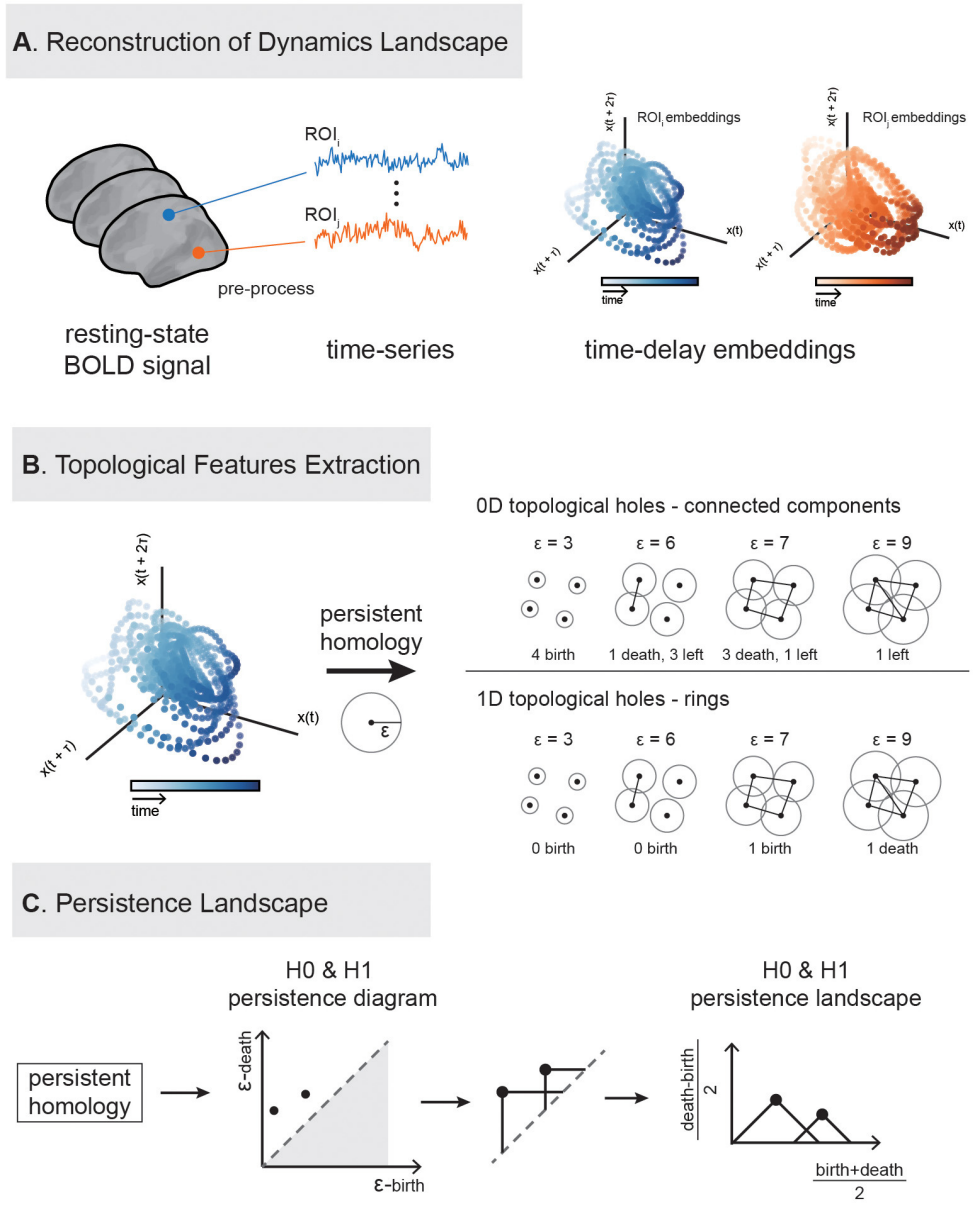
Flowchart of extracting topological features of brain activity using persistent homology. The analysis consists of three main steps. **(A)** Delay embedding construction: Delay embedding is applied to the original time series to reconstruct the system's state space. For illustration, we display two delay-embedding's in three dimensions. **(B)** Feature extraction: Two types of features (0-dimention and 1-dimenstion) are extracted from the embedded data. **(C)** Topological landscape construction: topological features were embedded into a computable space.

This study utilizes cortical functional activity data to extract persistent landscape features from each ROI via delay embedding and persistent homology, which are then applied to individual identification, behavioral prediction, and other analysis. The TDA analysis flowchart is illustrated in the Figure 1.

#### 2.2.1 Delay embedding

Delay embedding is a method used to reconstruct dynamical systems and is widely applied in non-linear dynamics analysis (Takens, 1981; Brunton et al., 2017; Raut et al., 2025). By using delay embedding, one can effectively reconstruct a one-dimensional time series into a high-dimensional state space, thereby capturing potential dynamical features (Figure 1A). The specific procedure of delay embedding requires determining two key parameters: embedding dimension and time delay. To ensure the optimal reconstruction of the dynamical system, this study uses the mutual information method to determine the optimal time delay and the false nearest neighbor method to determine the optimal embedding dimension (Kennel et al., 1992). After optimization using these methods, the best embedding dimension of 4 and time delay of 35 were determined for this experiment, and this parameter combination was used for subsequent topological analysis.

#### 2.2.2 Persistent homology

To extract topological features from the high-dimensional point cloud reconstructed through delay embedding of the time series, we performed 0-dimensional (H0) and 1-dimensional (H1) persistent homology analysis using the Giotto-TDA toolkit (Tauzin et al., 2021). Persistent homology is a tool for characterizing the topological structure of data from a multi-dimensional perspective, capable of identifying and tracking the appearance and disappearance of topological features such as connected components, loops, and cavities at different dimensions.

#### 2.2.3 Persistent landscape

We used the persistence landscape (PL), as proposed by Bubenik (2015), to describe the birth and death of topological holes in different dimensions. Given a point cloud dataset $X=\{x_{1},x_{2},\ldots ,x_{n}\}\subset R^{d}$, its topological structure across different spatial scales can be characterized by constructing a sequence of nested simplicial complexes. The most commonly used construction is the Vietoris–Rips complex: for a given distance threshold **ϵ > 0**, an edge is added between any pair of points whose Euclidean distance is < **ϵ**. More generally, a **k**-simplex (Supplementary Figure 1) is included whenever all pairwise distances among its **k+1** vertices are < **ϵ**.

As **ϵ** increases, the complex grows by progressively incorporating more simplices, leading to changes in its topological structure. Persistent homology tracks the “birth” and “death” of topological features (e.g., connected components, loops, and voids) over this filtration, generating persistence diagrams (PD) that summarize the multiscale topological organization of the data (Figure 1B). For a given topological feature, its persistence is defined as the difference between its death and birth times: **pers=d−b**.

To facilitate downstream statistical or machine learning analysis, we transform the PD into a PL, a functional representation that embeds topological features into a Hilbert space. Each feature **(b**_**i**_**, d**_**i**_**)** is mapped to a triangular tent function:


$${\text{Λ}}_{i}\left(t\right)={\left[min\left(t-b_{i},d_{i}-t\right)\right]}_{+}=max\left(0,min\left(t-b_{i},d_{i}-t\right)\right)$$

Then the persistence landscape is defined as the sequence of functions **{λ**_**k**_(**t**)**}**_**k**∈ℕ_, where **λ**_**k**_(**t**) is the **k**-th largest value of **{Λ**_**i**_(**t**)**}**_**i∈I**_ at each time **t**. These functions can be discretized into vectors for efficient statistical analysis.

#### 2.2.4 Experimental design and feature extraction

In this study, we performed persistent homology analysis at both H0 and H1 levels for each ROI of every subject. Here, the H0 features correspond to the evolution of connected components, while the H1 features capture the appearance and disappearance of loop structures. The specific steps are as follows: (a) Generate point clouds for each ROI using delay embedding of the fMRI time series. (b) Use the Giotto-TDA toolbox to compute 0-dimensional (H0) and 1-dimensional (H1) persistence diagrams for each ROI, capturing the birth and death of topological features (i.e., connected components and loops). (c) For each ROI's persistence diagram, compute the corresponding persistence landscape (PL) for both H0 and H1. Each landscape is compressed to a 100-dimensional vector. (d) All persistence landscapes are flattened and concatenated to obtain a subject-level topological feature vector. Each subject has 200 ROIs, and each ROI yields two persistence landscapes (one for H0 and one for H1), resulting in a total of **200 × 100 × 2=40000** topological feature vectors per individual (An example is shown in Supplementary Figure 2). In this way, we preserve the topological dynamic information of each ROI across different dimensions, providing a foundation for further statistical analysis or individual difference modeling.

### 2.3 Traditional time-series temporal features

To characterize the intrinsic temporal dynamics of fMRI signals, we extracted a set of canonical time-series features using the catch22 toolbox proposed by Lubba et al. (2019). The catch22 framework provides a collection of 22 time-series features that were selected through a large-scale, data-driven analysis to maximize classification performance while minimizing redundancy. These features capture a diverse range of temporal properties and have been validated across a wide variety of real-world datasets.

The temporal features from catch22 span multiple domains of time-series analysis, including but not limited to: Autocorrelation and predictability (e.g., first-lag autocorrelation, partial autocorrelation); Distributional properties (e.g., time reversibility, outlier measures); Non-linear and entropy-based features (e.g., sample entropy, fluctuation analysis); Periodicity and frequency content (e.g., periodicity score); Change-point and stationarity measures. For a complete list and mathematical definitions of the 22 features, we refer the reader to the original publication by Lubba et al. and the official catch22 documentation. All features were computed for each ROI independently based on the preprocessed fMRI time series.

In addition to the standard 22 features from catch22, we further included the mean and standard deviation (std) of each time series, resulting in a 24-dimensional feature vector for each region of interest (ROI).

This set of features serves as a conventional, interpretable baseline for modeling individual differences in brain dynamics, and provides a useful comparison point for evaluating the added value of topological representations such as persistent homology.

### 2.4 Validation of the “fingerprinting” effect

To validate whether the topological features exhibit stability and discriminability at the individual level, we adopt a fingerprinting analysis framework to evaluate their individual distinguishability (Finn et al., 2015). The core idea of this analysis is that if a certain feature shows consistency for the same subject across different scan sessions and has sufficient discriminative power within the population, it can be considered a 'topological fingerprint' for identifying individuals.

#### 2.4.1 Data division and matching process

The specific process is as follows: (a) Feature Set Construction: The topological features obtained from all subjects on day 1 are used as the database, denoted as $\left\{T_{1}^{\left(1\right)},T_{2}^{\left(1\right)},\ldots ,T_{N}^{\left(1\right)}\right\}$, where $T_{i}^{\left(1\right)}$represents the topological feature vectors of subject **i** on day 1. The topological features obtained from all subjects on day 2 are used as the target set, denoted as $\left\{T_{1}^{\left(2\right)},T_{2}^{\left(2\right)},\ldots ,T_{N}^{\left(2\right)}\right\}$, where $T_{i}^{\left(2\right)}$represents the topological feature vectors of subject **i** on day 2. (b) Matching Strategy: For each individual $T_{i}^{\left(2\right)}$in the target set, the Pearson correlation coefficient between its topological feature vector and those of all individuals in the database is computed:


$$\begin{array}{l} r_{ij}=corr\left(T_{i}^{\left(2\right)},T_{j}^{\left(1\right)}\right),\;\forall j\in \left\{1,2,\ldots ,N\;\right\} \end{array}$$

where **r**_**ij**_ represents the similarity between the target individual **i** and the database individual **j**. (c) Identity Prediction and Accuracy Calculation: The database individual j^*^=argmax_**j**_ **r**_**ij**_ that maximizes **r**_**ij**_ is selected as the predicted identity for the target individual **i**. If j^*^=i, meaning a correct match, it is considered a correct identification. Finally, by calculating the ratio of correctly identified individuals to the total number of individuals, the identification accuracy is obtained. This is used to assess the strength of the topological feature's fingerprinting effect.

#### 2.4.2 Evaluation of multidimensional topological features

To systematically evaluate the contribution of different topological dimensions to fingerprinting performance, we perform matching tests using the following two types of features: 0-dimensional features (connected components) and 1-dimensional features (loops), to comprehensively reflect the topological structure of individuals. By comparing the identification accuracy under these two types of features, we can reveal the contribution of topological information from different dimensions to individual stability and discriminability, providing theoretical support for subsequent studies on individual differences. Beyond the whole-brain ROI-level fingerprint analysis, we further quantified inter-network differences in fingerprint effects by analyzing topological features extracted from ROIs within individual networks.

### 2.5 CCA modeling of topological features and behaviors

To investigate the relationship between individual subjects' topological features and behavioral measures in a single holistic multivariate analysis, we carried out canonical correlation analysis (CCA) (Smith et al., 2015; Liu et al., 2023). H0 and H1 are integrated together as topological features, or, as PL features (**1013** **subjects×40000** **features**). We followed the canonical correlation analysis (CCA) pipeline described by Smith et al. Specifically, we used 478 behavioral measures from the HCP dataset, applied filtering procedures to exclude variables that did not meet the inclusion criteria (resulting in 147 remaining variables), and regressed out nuisance variables to account for potential confounds. To mitigate overfitting, principal component analysis (PCA) was applied to reduce the dimensionality of both the topological features matrix and the behaviors matrix to 100 components each. Nonparametric permutation testing with 10,000 iterations was conducted to evaluate statistical significance.

To quantitatively compare the contributions of topological features from different ROIs to the significant CCA mode, we separated the H0 and H1 features for each ROI. For each individual ROI, we performed principal component analysis (PCA) on the topological features and retained the first principal component as the representative feature for that ROI in H0 (or H1), resulting in a dimensionality reduction from 1, 013 *subjects*× 100 to 1013 *subjects*× 1.

### 2.6 Individual traits prediction and comparison

To evaluate whether topological features can effectively represent individual differences and compare them with traditional time series features, we designed classification and regression tasks. In the topological features set, both H0 and H1 features are used together for the classification task and the regression task.

#### 2.6.1 Gender classification task

For the input features, we compare the performance between PL topological features and catch22 temporal features. To ensure fairness in comparison, we performed PCA on both feature sets, reducing them to 100 dimensions. This resulted in a unified feature matrix (**nSub×100**), which was used for downstream individual identification modeling. We refer to the resulting 100-dimensional representations as the topological features (for PL) and temporal features (for catch22), respectively.

To evaluate the predictive power of different feature representations for individual gender classification, we compared 24 selected temporal features from Catch22 and topological features derived from persistent homology. We employed a logistic regression classifier in a five-fold cross-validation framework. For each feature type, class probabilities were predicted across folds, and receiver operating characteristic (ROC) curves were constructed based on the aggregated prediction scores. The area under the ROC curve (AUC) was used as the primary metric to quantify classification performance.

#### 2.6.2 Behavior regression task

Furthermore, we utilized the categorized behavioral traits provided by the HCP dataset. Specifically, we included six categories of behavioral measures: Cognition (52 items), Emotion (24 items), Personality (5 items), Psychiatric and Life Function (30 items), Sensory (13 items), and Substance Use (69 items). For each category, we performed principal component analysis (PCA) and extracted the first principal component, which served as a summary score representing the major dimension of variation within that behavioral domain. We conducted a separate analysis of the Penn Matrix Reasoning Test (PMAT24_A_CR) due to its recognized significance in assessing fluid intelligence (Schirner et al., 2023). Detailed item lists and corresponding PCA loadings are provided in Supplementary Table 1.

A linear regression model was used to predict each behavioral summary score based on either topological features or catch22 temporal features. Model performance was evaluated using five-fold cross-validation, repeated 10 times with different random splits for permutation testing. In each fold, we computed the Pearson correlation coefficient (*r*) between the predicted and observed behavioral scores. Finally, we conducted a paired *t*-test to compare the prediction performance of the topological features and the catch22 temporal features across all repetitions and behavioral categories.

## 3 Results

### 3.1 Topological features enable reliable individual identification across sessions

**Figure 2:**
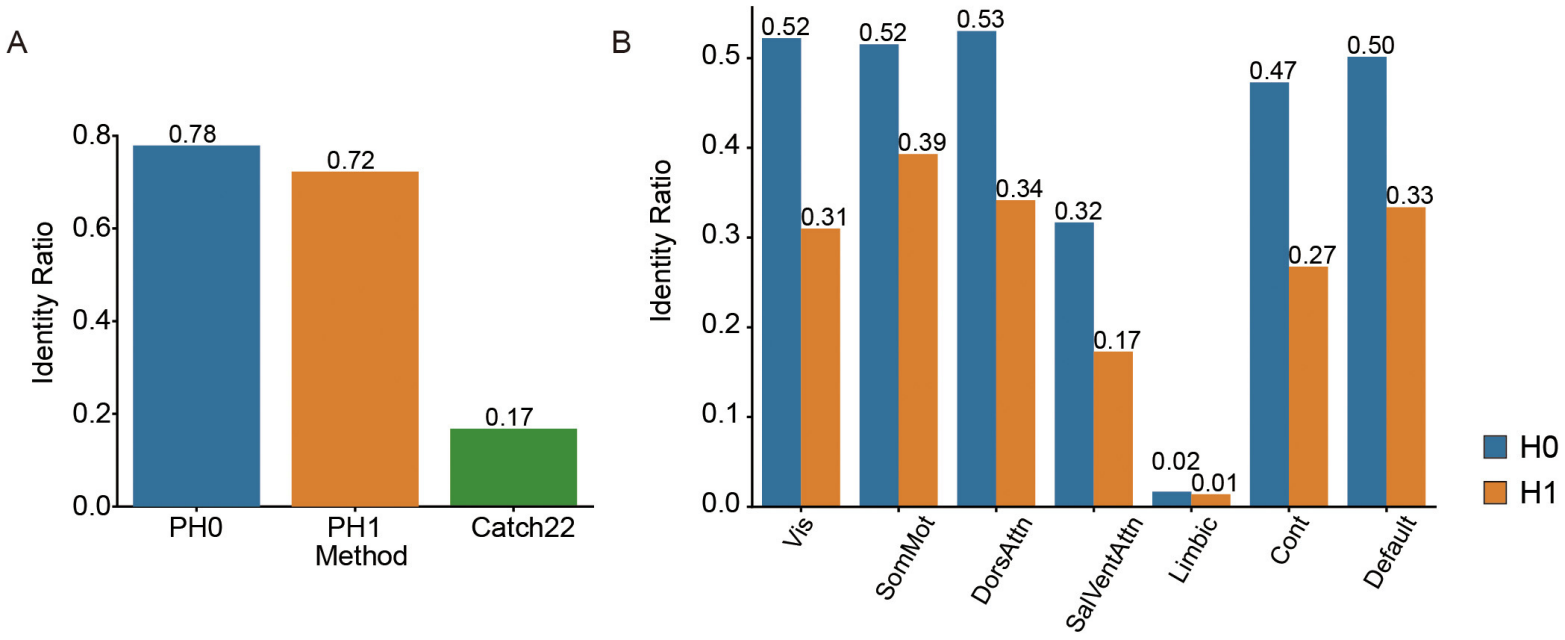
Cross-session identification accuracy of individual topological features. **(A)** Identification accuracy (“fingerprinting effect”) across sessions using three methods: persistent homology in dimension 0 (H0), dimension 1 (H1), and a standard time-series feature set (Catch22). **(B)** Identification accuracy of H0 and H1 features across seven functional networks: Visual (Vis), Somatomotor (SomMot), Dorsal Attention (DorsAttn), Salience/Ventral Attention (SalVentAttn), Limbic, Control (Cont), and Default Mode.

We first exam whether our proposed topological features can serve as an individual signature. We test the identity ratio (“fingerprinting effect”) between 2 days scan (See Method). The accuracy of successful individual identification is compared across three feature types: H0, H1, and Catch22. Both H0 and H1 yielded high fingerprint values (0.78 and 0.72, respectively), while Catch22 produced a substantially lower fingerprint value (0.17), suggesting that topological features (H0 and H1) are more effective for capturing individual-specific patterns than the time-series feature set (Figure 2A).

These results demonstrate that even in large-scale samples with over a thousand participants, the topological structural features of individuals maintain high stability and discriminability across different scan sessions, exhibiting good cross-time individual consistency and providing a foundation for individual-level representation and modeling.

Fingerprinting effects varied across different functional networks, indicating network-specific differences in individual identifiability. H0 yielded consistently higher fingerprint values than H1 across all networks (Figure 2B). The Visual, Somatomotor, and Dorsal Attention networks showed the highest fingerprint values under both conditions, with H0 values around 0.52–0.53 and H1 values ranging from 0.31 to 0.39. In contrast, the Salience/Ventral Attention, Limbic, and Control networks showed relatively lower fingerprint values.

### 3.2 Topological features outperform temporal features in gender classification

To further assess the discriminative power of the extracted features, we evaluated the performance of topological features and temporal features in predicting individual gender. A logistic regression classifier was trained on each feature type using data from 543 female and 470 male participants. Receiver operating characteristic (ROC) curves were computed to quantify classification performance.

**Figure 3:**
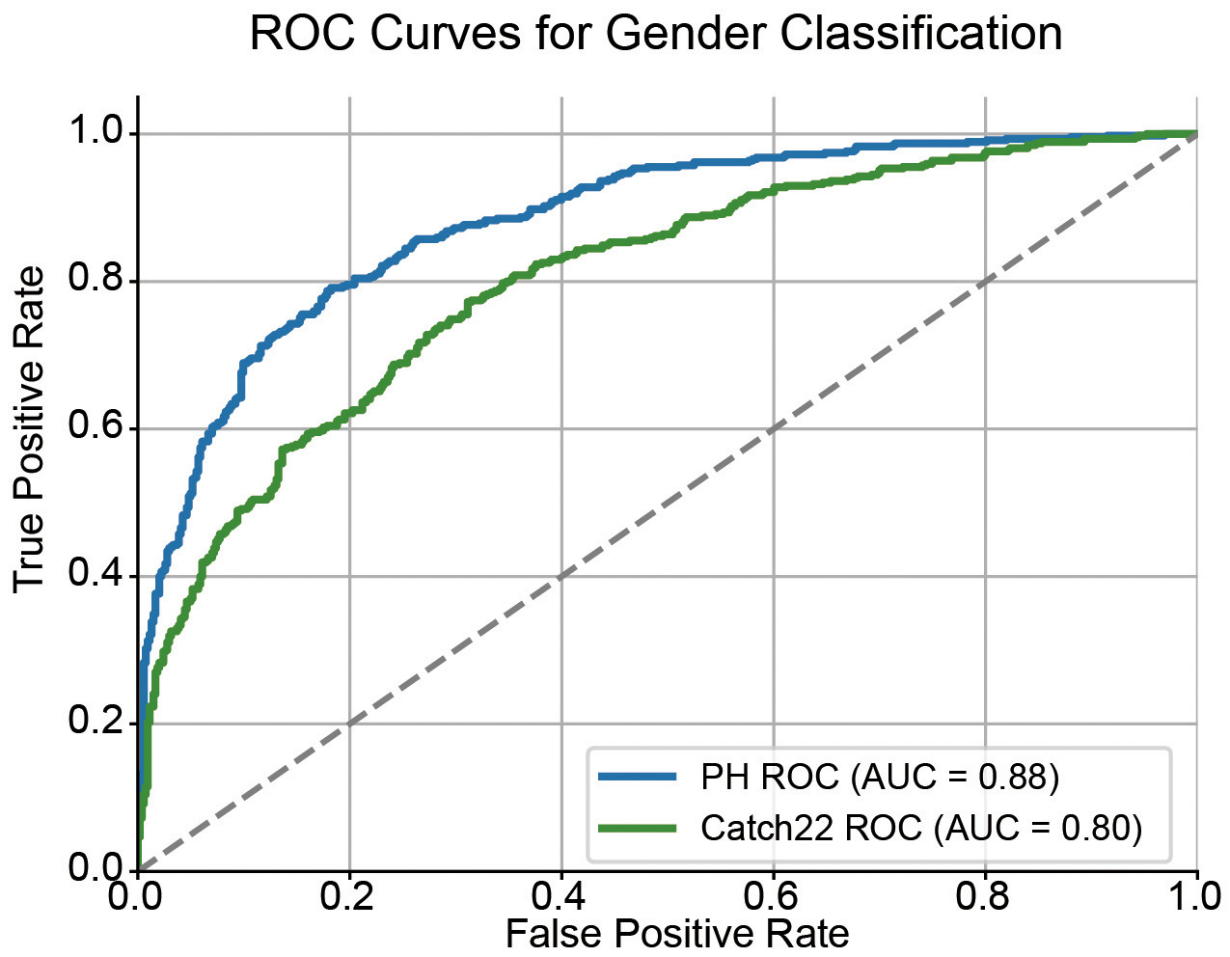
ROC curves for gender classification using topological and conventional time-series features. Receiver Operating Characteristic (ROC) curves compare the performance of persistent homology (PH) features and Catch22 time-series features in classifying gender. The diagonal dashed line represents the chance level (AUC = 0.5).

As shown in Figure 3, the topological features achieved an AUC of 0.88, while the temporal features yielded an AUC of 0.80. These results indicate that the topological features capture more discriminative information related to gender than conventional time-series features, suggesting that the non-linear and geometrical structure encoded in topological features provides a more informative characterization of individual brain dynamics.

### 3.3 Topological features reveal significant brain-behavior covariation

**Figure 4:**
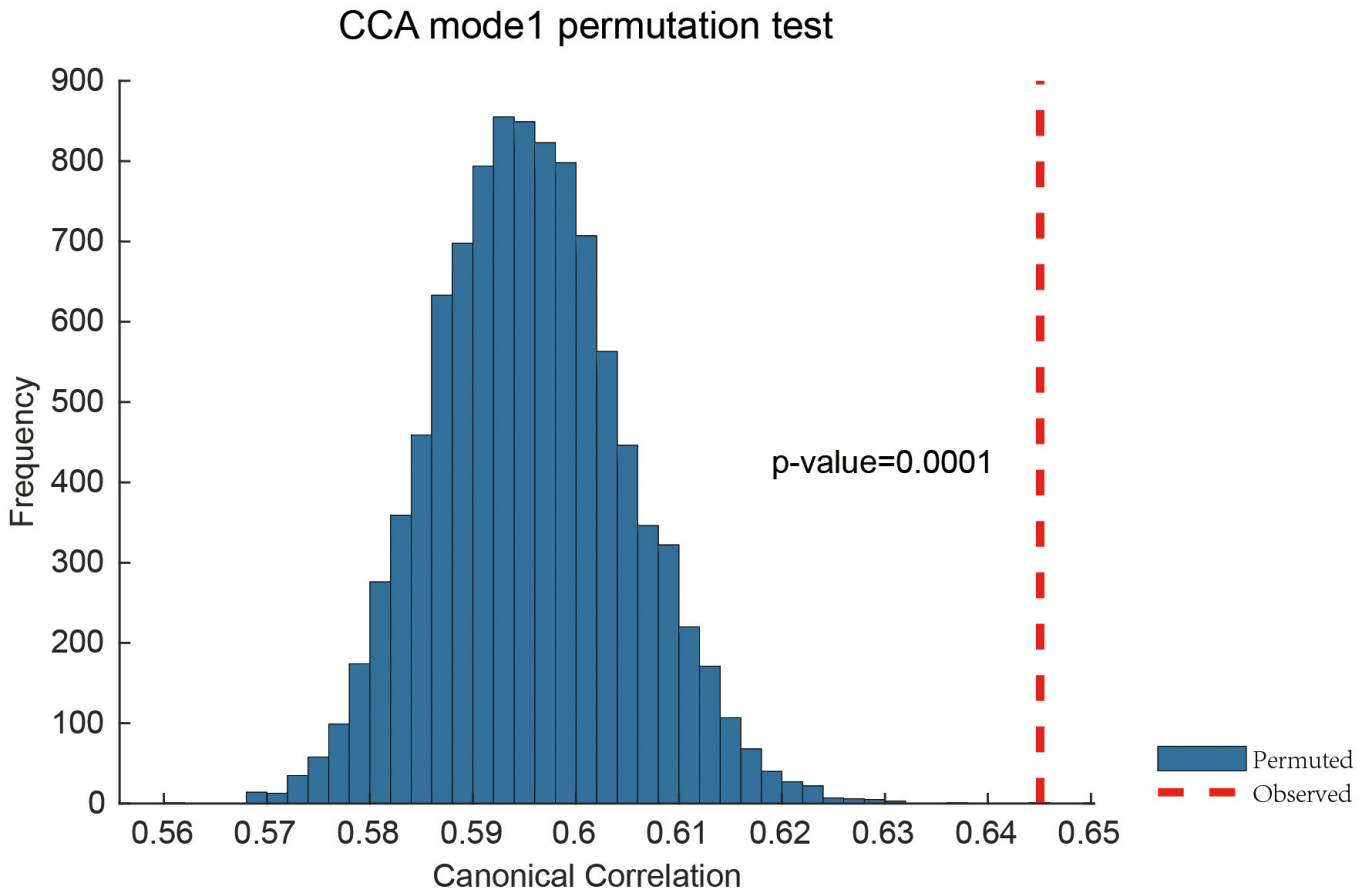
Permutation validation of Canonical Correlation Analysis. The permutation test with 10,000 iterations was performed. Only one significant brain-behavior covariance pattern was identified, with a correlation value (red vertical line) of 0.6451 between brain topological features and behavioral measurements.

We identified a significant mode of covariation between topological brain features and behavioral traits using canonical correlation analysis (CCA), with the first canonical correlation reaching *r* = 0.6451 and a permutation-based *p*-value of 0.0001 (Figure 4). This result confirms that the extracted topological features carry meaningful information about inter-individual behavioral variation.

#### 3.3.1 Network-specific topological contributions characterize the brain-behavior association

**Figure 5:**
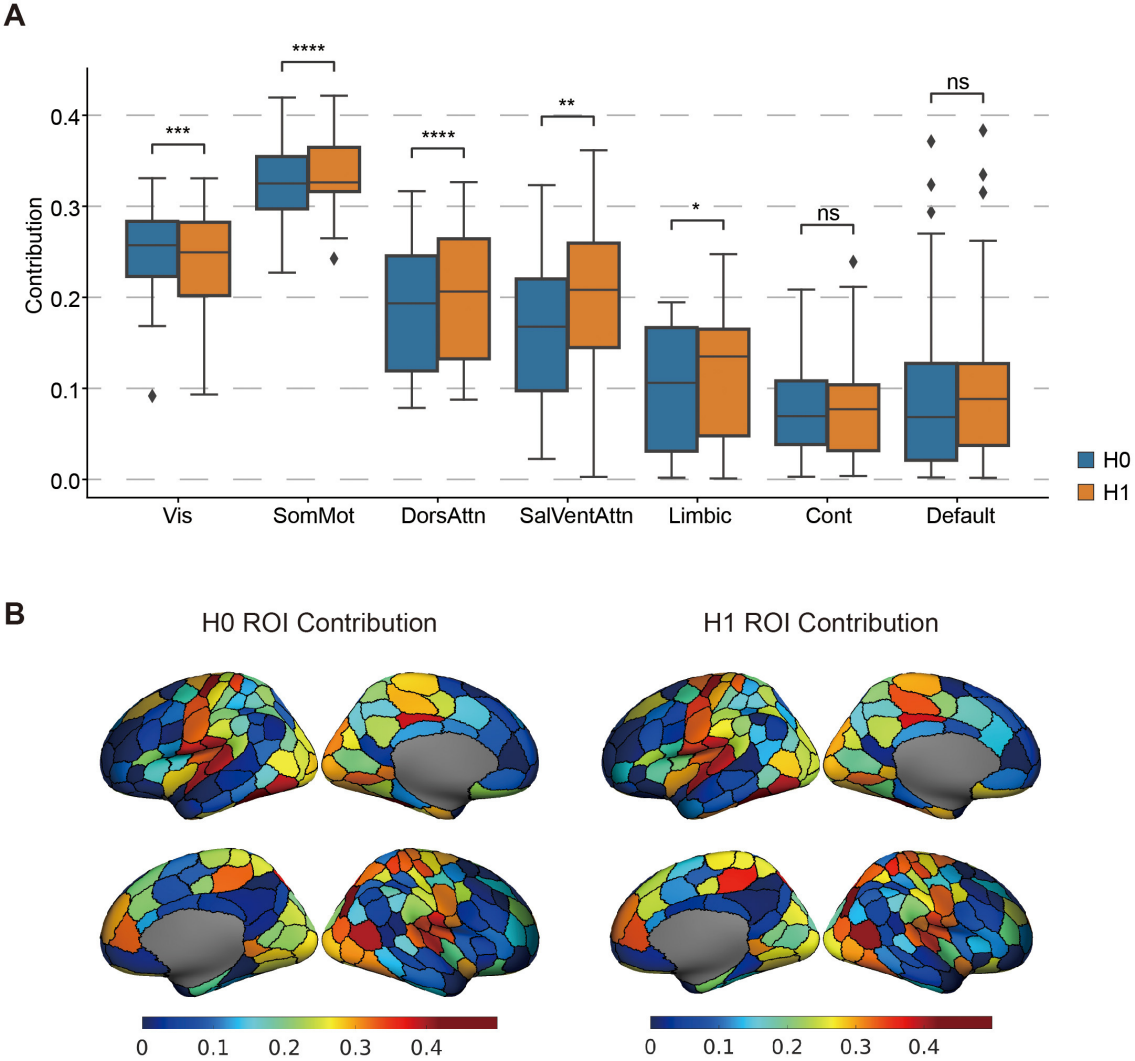
The Significant brain regional CCA weight pattern. **(A)** The contribution of the topological features of different networks to the covariance. The *t*-test compared the differences in network contributions between H0 and H1. *P* < 0.05*, *p* < 0.01**, *p* < 0.005***, *p* < 0.0001****, ns, non-significant. **(B)** Maps of H0 ROI contribution and H0 ROI contribution. The color bar represents the CCA weights.

To further interpret this brain-behavior mode, we analyzed the regional contributions of topological features across canonical networks. As shown in Figure 5A, the mode was characterized by strong positive contributions from the sensorimotor, visual, and dorsal attention networks, while the control, default mode, and limbic networks showed relatively lower contributions. This spatial pattern is further visualized in Figure 5B, which presents surface maps of ROI-level contributions for both H0 and H1 features. This finding is consistent with prior work by Santoro et al., which demonstrated network-specific effects in topological analyses of brain dynamics (Santoro et al., 2024).

Although H0 and H1 landscapes exhibit broadly similar patterns across the cortex, significant differences emerge across specific functional networks. Notably, H0 features contribute more strongly within the visual network, whereas H1 features dominate in the sensorimotor and dorsal attention networks. These differences suggest that distinct topological structures (i.e., components vs. loops) may underlie functional specialization in different brain systems. Taken together, these results highlight that persistent homology captures meaningful and functionally organized variation in brain activity, supporting its utility for uncovering network-level structure in brain-behavior relationships.

#### 3.3.2 CCA mode reflects cognition and psychopathological risk

**Figure 6:**
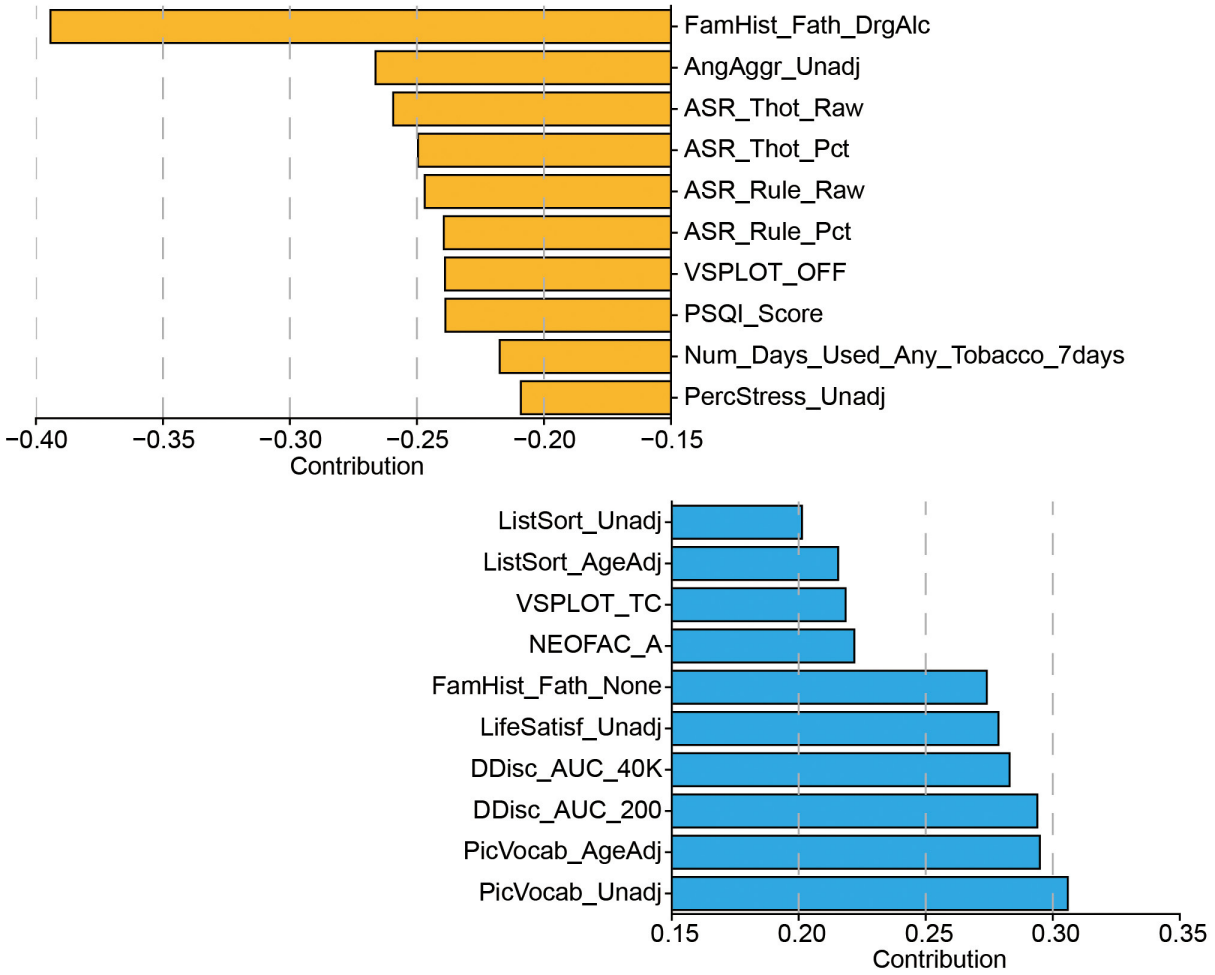
The set of behavioral measurements most strongly associated with the CCA mode of population variability. Display only the top ten behaviors corresponding to the CCA weights.

To further interpret the behavioral dimension associated with the first CCA mode, we examined the individual behavioral items with the highest absolute contributions (Figure 6). Interestingly, we observed a clear dissociation between positive and negative contributors, revealing a meaningful latent structure. The positively weighted behaviors were primarily associated with cognitive functions, including measures of working memory (ListSort_Unadj, ListSort_AgeAdj), visuospatial processing (VSPLOT_TC), vocabulary knowledge (PicVocab_Unadj, PicVocab_AgeAdj), personality traits (NEOFAC_A), and decision-making performance (DDisc_AUC_200, DDisc_AUC_40K). These findings suggest that individuals with higher topological scores along the first CCA mode tend to exhibit higher cognitive functioning and more adaptive personality traits.

In contrast, the negatively weighted behaviors were dominated by items related to psychopathology and behavioral risk, particularly those from the Adult Self-Report (ASR) and psychosocial domains. These include elevated scores on thought problems (ASR_Thot_Raw, ASR_Thot_Pct), rule-breaking behavior (ASR_Rule_Raw, ASR_Rule_Pct), aggression (AngAggr_Unadj), perceived stress (PercStress_Unadj), and poor sleep quality (PSQI_Score), as well as history of parental substance abuse (FamHist_Fath_DrgAlc) and recent tobacco use. Together, these patterns indicate that the canonical axis identified by CCA reflects a behavioral gradient spanning from cognitive strength and psychological well-being (positive end) to externalizing symptoms, stress, and substance use risk (negative end). This further supports the interpretability and behavioral relevance of the topological features in capturing meaningful individual differences.

### 3.4 Behavioral relevance of topological vs. temporal features

To further investigate the relationship between topological brain features and different domains of behavior, we conducted a set of linear regression analyses targeting six behavioral categories defined by the HCP dataset: Cognition, Emotion, Personality, Psychiatric and Life Function, Sensory, and Substance Use. For each category, the first principal component of behavioral items was used as the target variable, and prediction performance was quantified using the Pearson correlation coefficient (Supplementary Table 2).

Interestingly, across multiple domains—particularly Cognition, Emotion, and Personality—the predictive performance of topological features significantly exceeded that of the temporal features. For example, in the fluid intelligence domain, topological features achieved a correlation of *r* = 0.17 ± 0.012, compared to *r* = 0.15 ± 0.011 for temporal features, although they achieve similar correlation in total cognition (*r* = 0.27 vs. *r* = 0.28). Similar patterns were observed for emotion (*r* = 0.09 vs. *r* = 0.00), personality (*r* = 0.12 vs. *r* = −0.02), psychiatric and life function (*r* = 0.10 vs. *r* = 0.07) and substance use (*r* = 0.20 vs. *r* = 0.15), with differences reaching high statistical significance.

In contrast, for the Sensory category, catch22 features showed slightly better predictive power (*r* = 0.05 ± 0.014) than PL features (*r* = 0.04 ± 0.018).

These findings suggest that topological features derived from persistent homology are more strongly associated with higher-order psychological and cognitive traits, potentially reflecting global or structured patterns in brain dynamics relevant to complex behaviors. In contrast, low-level or externally driven behaviors, such as sensory, may be more closely related to local or low-order temporal features captured by traditional time-series metrics.

## 4 Discussion

Traditional functional connectivity analyses condense fMRI time-series into static correlations between brain regions, but this approach overlooks rich temporal dynamics. By analyzing fMRI from a temporal perspective—using delay embedding to reconstruct the trajectory of each region's activity—we capture information that static connectivity alone cannot (Anderson et al., 2018). This dynamical-systems view treats the brain as an evolving trajectory in a high-dimensional state space, allowing us to characterize patterns like recurrent oscillations or transitions that are invisible to static correlation measures. Our findings underscore the value of this approach: the persistent homology features extracted from time-delay embedding's encode distinct aspects such as cognition and personality. In essence, the temporal structure of neural signals contains meaningful signatures of brain function that complement and enrich conventional connectivity analyses. This supports a growing recognition in network neuroscience that brain function is inherently dynamic and that temporal features (e.g., variability, transitions, recurrences) can illuminate individual traits and states in ways static functional connectivity cannot.

Our results show that the proposed framework effectively extracts meaningful and stable representations from fMRI time-series. The derived persistent landscape (PL) features, whether from H0 or H1, serve as reliable individual “fingerprints”, capturing distinctive and reproducible patterns in each person's brain dynamics—similar to how static connectomes uniquely identify individuals (Finn et al., 2015). These features are robust to noise, summarizing each ROI's trajectory across multiple scales in a compact, information-rich form. Importantly, Topological features also carry biological relevance: they enable accurate gender classification from resting-state data, reaching accuracy comparable to traditional connectivity-based methods (Zhang et al., 2018). Unlike network-based features that rely on predefined assumptions about inter-regional connectivity, our approach employs persistent homology to extract topological features directly from time-series data, enabling the discovery of meaningful individual differences without the constraints of inter-regional assumptions. Another strength of our approach lies in its multiscale nature and robustness to variations in image acquisition parameters. Persistent homology analyzes data across a continuous range of thresholds, capturing the entire evolution of topological features—from their birth to their death (Kumar et al., 2023).

Our study suggests that, in multivariate behavioral analyses, higher-order topological features (H1) tend to exhibit greater canonical weights, potentially indicating that more complex brain topology may provide additional information for explaining individual differences, particularly in several specific cognition. These H1 features, reflecting recurrent or cyclic patterns in ROI activity, were strongly linked to a brain–behavior mode identified via CCA, suggesting that sustained loop interactions carry richer behavioral relevance than simple connected components (H0). This aligns with recent findings that higher-order topological structures, such as cycles or simplicial motifs, better capture individual fingerprints and cognitive variance than traditional pairwise metrics (Expert et al., 2019; Santoro et al., 2024). Our CCA uncovered a dominant mode linking PL features (both H0 and H1 features) to both cognitive performance and socio-emotional traits (e.g., ASR measures), supporting the idea that brain dynamics integrate cognitive and affective processes (Simon et al., 2020). This interpretation finds some support in prior literature. Saggar et al. (2018, 2022), for instance, showed that simplifying fMRI data to a topological backbone still preserved correlations with task performance, implying that the relevant information for behavior was encoded in the core dynamic topology. Our results follow a similar logic: they highlight that individuals with different cognitive/personality profiles have measurably different topological “signatures” in how their brain activity evolves over time. Notably, the most behaviorally relevant topological signatures emerged in unimodal sensory networks—especially visual and somatomotor regions—highlighting their key role in anchoring individual variation. While these regions are often overlooked in favor of higher-order association networks, recent work suggests they exhibit strong between-subject variability and may interface with transmodal hubs to shape individual-specific cognition and emotion (Santoro et al., 2024). Topological features alone did not show strong predictive power for sensory in separate behavioral prediction analyses, the CCA pattern of the cortex potentially support a hypothesis that the dynamic structure of time series may facilitate the integration of information across unimodal and transmodal regions. Together, these findings underscore the value of topological analysis in revealing how complex, recurrent brain dynamics—especially within sensory systems—encode stable traits of thought, feeling, and behavior.

While our framework is promising, several limitations must be noted. Persistent homology (PH) features, such as birth and death times, are abstract and lack direct neurophysiological interpretation, making biological conclusions speculative without further validation. PH also operates at a high level of abstraction—distinct neural processes may yield similar topological signatures, potentially masking finer details. Our use of ROI-level signals may overlook sub-regional or frequency-specific dynamics, and parameter choices (e.g., embedding dimension) could affect sensitivity. Computational complexity may limit scalability to voxel-level or long time-series data. PH features should complement, not replace, conventional metrics, as some effects may be better detected by traditional methods. Finally, our findings—based on resting-state data in a specific cohort—may not generalize across populations or datasets, and test–retest reliability remains to be established.

In summary, we present a novel framework combining delay embedding and persistent homology to extract topological signatures from fMRI time-series, capturing individual-specific, biologically meaningful, and behaviorally relevant brain dynamics. Persistent landscape features act as stable neural fingerprints, enable accurate group classification (e.g., gender), and uncover a dominant brain–behavior mode linking topological features to cognitive and emotional traits. These findings highlight the value of topological analysis as a dynamic, multiscale complement to traditional connectivity metrics. Future work should explore task-based and clinical applications, improve interpretability through modeling, and integrate topological with conventional features. Extending analyses across spatial and temporal scales (e.g., voxel-level or sliding-window PH) may further reveal how brain topology evolves with state or condition. Our results advocate for broader use of TDA in neuroimaging, offering new tools to understand and track individual differences in brain function.

## Data Availability

The dataset analyzed during the current study is available in the Human Connectome Project repository (http://www.humanconnectome.org/). The Topological Data Analysis (TDA) toolkit used in this study can be accessed at https://github.com/giotto-ai/giotto-tda.
