# Supplementary figures and images for: Topological signatures of brain dynamics: persistent homology reveals individuality and brain–behavior links
Source: Front Hum Neurosci. 2025 May 30;19:1607941. doi: 10.3389/fnhum.2025.1607941 (PMC12163041; doi:10.3389/fnhum.2025.1607941)

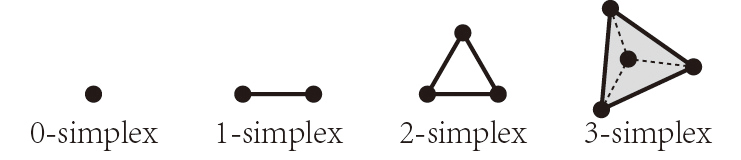

Supplement: Supplementary file 4 [file Image_1.jpeg]

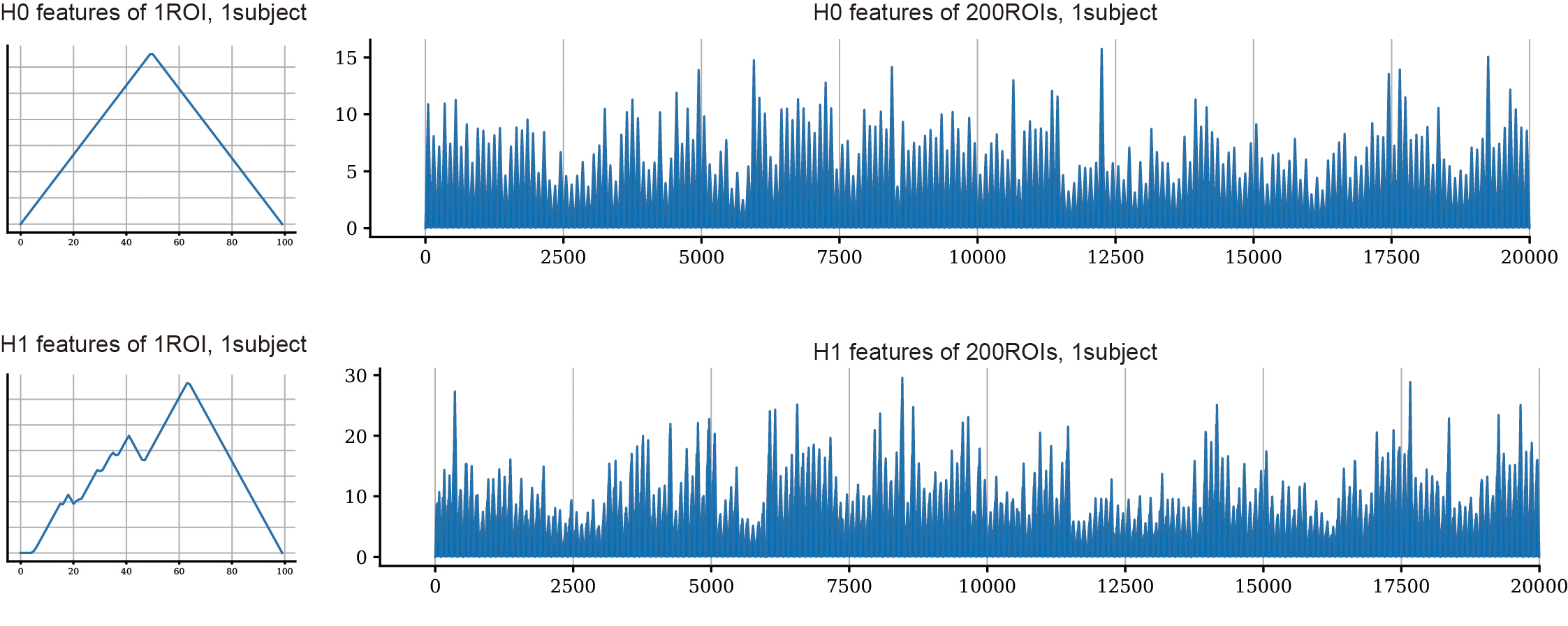

Supplement: Supplementary file 5 [file Image_2.jpeg]
